# Development and validation of an ELISA for a biomarker of thyroid dysfunction, thyroid peroxidase autoantibodies (TPO-Ab), in dried blood spots

**DOI:** 10.1186/s40101-020-00228-8

**Published:** 2020-07-16

**Authors:** Geeta N. Eick, Tara J. Cepon-Robins, Maureen J. Devlin, Paul Kowal, Larry S. Sugiyama, J. Josh Snodgrass

**Affiliations:** 1 Department of Anthropology, University of Oregon, Eugene, OR 97403 USA; 2 Department of Anthropology, University of Colorado Springs, Colorado Springs, CO 80918 USA; 3 Department of Anthropology, University of Michigan, Ann Arbor, MI USA; 4 University of Newcastle Research Centre for Generational Health and Ageing, Newcastle, New South Wales Australia; 5 Chiang Mai University Research Institute for Health Sciences, Chiang Mai, Thailand

**Keywords:** In-house immunoassay, Thyroid peroxidase autoantibodies, Dried blood spots, Old Friends Hypothesis

## Abstract

**Background:**

The prevalence of allergic and autoimmune conditions has been steadily increasing in wealthy nations over the past century. One hypothesis put forward to explain this is the Old Friends Hypothesis, which posits that increased hygiene, urbanization, and lifestyle changes have reduced our exposure to parasites and microbes that we co-evolved with, resulting in immune dysregulation. However, research in traditionally living populations, who are exposed to greater parasite and pathogen loads such as those encountered during our evolution, is limited, in part due to a lack of minimally invasive, field-friendly biomarkers of autoimmune disorders. We therefore developed an ELISA to assess positivity for thyroid peroxidase autoantibody (TPO-Ab), an indicator of autoimmune thyroid disease, based on dried blood spot (DBS) samples.

**Results:**

We used the Accubind anti-thyroid peroxidase test system to screen our validation samples comprising matched fingerprick DBS, venous DBS, and plasma samples from 182 adults. After confirming that we had TPO-Ab-positive individuals in our validation sample (*n* = 12), we developed an indirect ELISA to measure TPO-Ab levels from one 3-mm DBS punch. The sensitivity and specificity of our assay for DBS samples ranged from 91.7–100% and 98.2–98.8%, respectively, using a cut-off value of ≥ 26 IU/mL. Intra-assay reliability for duplicate quality control DBS punches was 5.2%, while inter-assay reliability ranged from 11.5–24.4% for high, medium, and low DBS controls. Dilutional linearity ranged from 80 to 120%, and spike and recovery experiments indicated that the DBS matrix does not interfere with the detection of TPO-Ab. TPO-Ab levels remained stable in DBS samples stored at − 28 °C or − 80 °C, but decreased over time in DBS samples kept at 22 °C or at 37 °C.

**Conclusions:**

We developed an in-house, kit-independent indirect ELISA assay to determine individuals’ TPO-Ab positivity based on dried blood spots, representing a cost-effective method with potential applications in a range of research settings.

## Background

The Old Friends Hypothesis (formerly “Hygiene Hypothesis”) [1] proposes that decreased exposure to infectious and parasitic agents during development is responsible for later immune dysregulation, chronic inflammation, and the increasing prevalence of allergic and autoimmune conditions [2]. Regular exposure to parasites and infectious disease-causing organisms throughout human evolution was likely the norm, but now increased hygiene, urbanization, and other relevant lifestyle changes have reduced this exposure in many populations around the world. While indirect testing of the Old Friends Hypothesis has been conducted in traditionally living populations by assessment of biomarkers of inflammation in dried blood spot (DBS) samples, direct testing with autoimmune and parasitic burden data has been mostly limited to studies conducted in wealthy nations [3]. However, populations from wealthy nations do not experience the same levels of infectious/parasitic exposure as more traditionally living populations [4]. Although biological anthropologists are ideally positioned to test the Old Friends Hypothesis because of their frequent work with both traditionally living populations and populations in early stages of market integration, the lack of minimally invasive, field-friendly biomarkers of autoimmune disorders has limited relevant research.

Autoimmune thyroid disorders (AITDs) are of special interest to biological anthropologists because of the importance of the thyroid gland in physiological mechanisms related to circumpolar adaptations among northern populations. Cold-adapted populations upregulate basal metabolic rates for increased internal heat production via elevations in thyroid hormone production [5–7]. This upregulation may work to prematurely exhaust the thyroid gland, resulting in higher rates of AITDs among these populations [8]. Combined with altered lifestyles related to the Old Friends Hypothesis, the ability to monitor AITD prevalence in these populations is crucial for understanding biocultural relationships and adaptive tradeoffs related to indigenous health.

Here, we describe the development of an ELISA assay to measure a biomarker of thyroid autoimmunity from dried blood spots (DBS). Thyroid gland dysfunction (hypothyroidism and hyperthyroidism) is a common condition worldwide, particularly among women in whom it can occur at any time in life, and AITDs are the major cause of thyroid dysfunction in iodine-replete populations [9]. Thyroid peroxidase is a membrane-associated enzyme expressed only in thyrocytes that catalyzes the oxidation of iodide on tyrosine residues in thyroglobulin for syntheses of the thyroid hormones T3 (triiodothyronine) and T4 (thyroxine). In thyroid autoimmune disorders such as Hashimoto’s thyroiditis (hypothyroidism) and Graves’ disease (hyperthyroidism), autoantibodies against TPO are produced (TPO-Ab), with the access of immune cells to TPO thought to be due to destruction of thyrocytes [10]. In addition to the deleterious effect of thyroid autoantibodies on the thyroid itself, TPO-Ab can fix complement and act as competitive inhibitors of several enzymes related to TPO [11], increase oxidative stress [12], and adversely impact several parameters of reproductive health, such as spermatogenesis, fertilization, and embryo quality, although the underlying pathological mechanisms for this are currently unknown [13].

Dried blood spots, created by collecting a small amount of blood from a fingerprick made with a lancet on filter paper cards such as Whatman 903 protein saver cards, offer logistical, shipping, and biosafety advantages in addition to low cost and analyte stability compared to venous blood samples, and are therefore the minimally invasive sample type of choice of many biological anthropologists [14]. Although many more biomarker assays have been developed for serum or plasma samples than for DBS, collection of serum or plasma samples requires a trained phlebotomist, centrifugation to separate the red blood cells from the serum/plasma fraction, and long-term freezer storage facilities if the samples are not going to be processed within a few hours, in addition to participants willing to undergo an invasive procedure (insertion of a needle into an arm vein). Although a TPO-Ab assay was previously validated for DBS by Hofman et al. (2004) [15], the commercial ELISA kit these authors validated (KRONUS Kalibre ELISA TPOAb kit, KR7260) was discontinued several years ago and, to the best of our knowledge, no other DBS assay for TPO-Ab currently exists.

We therefore developed and validated an ELISA to assess whether an individual is positive or negative for TPO-Ab based on a single 3-mm punch from a DBS.

## Methods

### Samples

Validation samples comprised matched fingerprick DBS (fDBS), venous DBS (vDBS), and plasma samples (“Eugene200 Validation Set” or E2V2) collected from a convenience sample of 182 adults (M to F 76:106; ≥ 18 years) from the Eugene/Springfield, Oregon (USA) area between November 2014 and February 2015 as described in detail previously [16]. IRB approval for this study was obtained from the Committee for the Protection of Human Subjects, University of Oregon (protocol # 7062016.007), and informed consent was obtained from all participants.

### Establishment of TPO-Ab levels in E2V2 plasma samples

To assess TPO-Ab levels in our plasma samples for validation of the in-house ELISA and to ensure that we had some TPO-Ab-positive individuals in our study population, we used the Accubind anti-thyroid peroxidase test kit (Cat. #1125-300) from Monobind Inc. (Lake Forest, CA 92630). This is a colorimetric ELISA for the quantitative detection of thyroid peroxidase autoantibodies in human serum or plasma samples that BioRad uses to assess TPO-Ab concentrations in their Liquichek Speciality Immunoassay controls, and we therefore treated the plasma TPO-Ab values obtained with this kit as “gold standard” values for the purposes of this validation. ELISAs were run according to the manufacturer’s instructions using a Biotek 405LS plate washer and EL808 plate reader. Plasma TPO-Ab values > 40 IU/mL using this kit are considered positive for the presence of anti-TPO autoantibodies.

### Liquid quality controls for the Accubind anti-thyroid peroxidase test system

Liquichek Speciality Immunoassay Control Levels 1–3 (Lot# 60223) were run on each Accubind ELISA plate as quality controls. The TPO-Ab levels assigned to these controls were the averages of the Monobind AccuBind ELISA and Monobind AccuLite CLIA values specified on the BioRad eInserts for Lot# 60223.

### DBS quality controls for the in-house assay

Three sets of quality control DBS were made by combining equal amounts of Liquichek Speciality Immunoassay controls 1, 2, and 3 with packed red blood cells obtained as described in detail in Eick et al. (2019). Fifty microliter aliquots of these mixtures were then pipetted out on Whatman 903 filter paper cards to create the quality controls that were stored at − 28 °C.

### Detection of TPO-Ab in dried blood spots

To determine if the measurement of TPO-Ab levels in DBS was feasible prior to investing time and resources into developing an in-house assay, 3-mm punches from the QC DBS cards were eluted in 250 μl of Accubind sample diluent and these DBS sample eluates were run on an Accubind TPO-Ab ELISA plate.

### ELISA development (“in-house” assay)

We developed our own in-house indirect ELISA assay to measure TPO-Ab levels in DBS; note that this kit-independent assay can be run in any laboratory with access to basic ELISA equipment such as a plate reader and basic laboratory reagents. Nunc Maxisorp plates (Fisher Scientific, Cat. # 439454) were coated overnight at 4 °C with a 1 μg/mL solution of human thyroid peroxidase (Sino Biological Cat. #13194-H08B) in filter-sterilized (0.22 μm filter) 1X phosphate-buffered saline (PBS). DBS samples were eluted by adding 300 μl of sample diluent (1 X PBS + 1 mg/mL gelatin +1 mg/mL bovine gamma globulin +1 mg/mL bovine serum albumin +0.05% Tween-20) to a single 3-mm-diameter punch in a borosilicate culture tube, covering the tube with parafilm, and placing it in the fridge overnight. On the day of the assay, the coated plate was removed from the fridge and allowed to equilibrate to room temperature for 30 min. The coating solution was aspirated from the wells using an automated plate washer (Biotek 405 LS), and 200-μl blocking was then added to each well (1 X PBS + 1 mg/mL bovine gelatin). The plate was sealed with a plate sealer and left on the benchtop at room temperature for 1 h for blocking. The plate was then washed 3X with 350 μl wash buffer (1 X PBS + 0.05% Tween-20) after which 100 μl DBS eluent, 1:200 diluted plasma, or 100 μl of the standard was added in duplicate wells. After a 90-min incubation at room temperature on the benchtop, the plate was washed as described above and 100 μl of a 1:25,000 of HRP-labeled detection antibody (rabbit anti-human IgG, cat. #ab6759, Abcam) was added per well and the plate was incubated at room temperature for 1 h on the benchtop. The plate was then washed 5× with wash buffer, and 100 μl of TMB substrate (KPL TMB Microwell Peroxidase Substrate System) was added per well. The color was allowed to develop in the dark for 15 min, and then 50 μl of 0.5 M H_2_SO_4_ was added per well to stop the reaction. To ensure mixing, the plate was shaken on a plate shaker at ~300 rpm for 1 min, after which absorbance at 450 nm with wavelength correction at 630 nm was read on a Biotek plate reader (Biotek EL808). A four-parameter logistic curve was used to fit the absorbance reading of the standards, and concentrations of unknowns were read off this curve. DBS samples with absorbance values higher than the highest standard were rerun by eluting them in 900 μl diluent and multiplying the final concentration obtained by 3.

### Standards

Liquichek Speciality Immunoassay Control Level 3 (Lot# 60223) was used to create eight liquid standards that were run on each plate. The TPO-Ab level assigned to this standard was the average of the Monobind AccuBind ELISA and Monobind AccuLite CLIA values specified on the BioRad product insert. Serial 2-fold dilutions were made of the top standard (1/100 dilution of the Liquichek Speciality Immunoassay Control Level 3) to obtain 7 dilutions. The final standard (zero standard) comprised sample diluent only.

### Sensitivity and specificity of the in-house assay and determination of the DBS cut-off value for TPO-Ab positivity

TPO-Ab levels were measured in 182 E2V2 plasma samples using the in-house assay to determine the sensitivity and specificity of the in-house assay relative to Accubind results for plasma. The sensitivity and specificity of the tests and 95% confidence intervals (CIs) were calculated using a 2-by-2 contingency table. The sensitivity of an assay refers to the percentage of samples correctly identified as being positive, whereas specificity refers to the proportion of negative samples correctly identified as such. Levels of TPO-Ab were also assessed in all 182 E2V2 vDBS and fDBS samples from individuals with a plasma concentration higher than the lowest standard in the Accubind assay to determine the DBS cut-off value for TPO-Ab positivity and to assess the sensitivity and specificity of the in-house assay for DBS samples.

### Dilutional linearity

Three vDBS samples that were anti-TPO positive were diluted 1/2, 1/4, and 1/8, and percentage recovery relative to the undiluted sample was calculated.

### Spike and recovery

To assess the ability of the in-house ELISA assay to accurately diagnose an individual as TPO-Ab positive in the potential presence of interfering matrix components, a spike and recovery experiment was conducted. DBS punches (3 mm) from seven individuals negative for TPO-Ab (< 26 IU/mL) were pooled and eluted in 2100 μl elution buffer, and then, sufficient Liquichek Speciality Immunoassay control was added to increase the expected concentration of the spiked samples by 43 IU/mL. TPO-Ab levels of the spiked sample and elution buffer were then measured, in addition to the TPO-Ab level in the corresponding unspiked sample.

### Reliability: intra- and inter-assay coefficients of variation (CV)

Intra-assay CV for DBS was determined by punching out 36 vDBS spots in duplicate and running the eluates on a single plate. Inter-assay CV was determined by running the high-, medium-, and low-TPO-Ab DBS quality controls (QCs) on eight in-house plates.

### Limit of detection (LOD)

The limit of detection of the in-house TPO-Ab ELISA was calculated by adding two standard deviations to the mean of 17 blank wells containing assay buffer only run on a single plate and extrapolating the corresponding TPO-Ab concentration from the four-parameter logistic curve.

### Analyte stability in DBS

Punches from vDBS samples from four different individuals were stored at − 28 °C, room temperature, and 37 °C for 2, 7, 14, and 28 days to determine the stability of TPO-Ab concentrations to various storage conditions. TPO-Ab levels in these samples were expressed as a percentage of the TPO-Ab concentration in matched samples stored at − 80 °C that were thawed only for this assay. To assess the impact of the number of freeze-thaw cycles on TPO-Ab concentrations, vDBS samples from six individuals were thawed at room temperature 2, 4, 8, or 12 times and were refrozen after each thawing session at − 28 °C. All samples were thawed a final time for the assay for a total number of freeze-thaw cycles of 3, 5, 9, and 13, respectively. TPO-Ab levels in these samples were expressed as a percentage of the TPO-Ab concentration in matched samples stored at − 80 °C that were thawed only once.

## Results

### Establishment of TPO-Ab levels in plasma samples using the “gold standard” method

Of the 182 plasma samples in our validation set, 12 were positive for TPO-Ab (> 40 IU/mL) (6.6% of our sample population), 144 samples had undetectable levels of TPO-Ab (78.3%), and 28 samples (15.2%) had TPO-Ab levels between 1.25 and 40 IU/mL and are considered negative for TPO-Ab according to the Accubind Anti-TPO ELISA specifications (total negative percentage, 93.5%). Of the 12 participants positive for TPO-Ab, 10 were women (9.4% of all women participants) and two were men (2.6% of all male participants). Levels of the three Liquichek Speciality Immunoassay controls (Lot# 60223) were all within the ranges specified for the Accubind TPO-Ab ELISA on the BioRad insert for these controls (data not shown).

### Detection of TPO-Abs in dried blood spots using the Accubind TPO-Ab ELISA kit

**Fig. 1:**
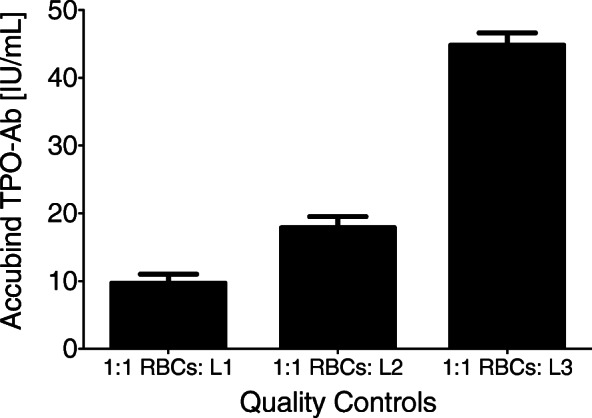
Thyroid peroxidase autoantibodies (TPO-Ab) are detectable by enzyme-linked immunoassay in 3-mm punches taken from dried blood spot samples. Washed red blood cells were spiked with Liquichek Speciality Immunoassy Control (levels 1, 2, or 3) and then dropped on Whatman 903 Protein Saver filter paper cards. Cards were dried for 4 h and then frozen at − 80 °C. Levels of TPO-Ab in one 3-mm punch taken from these cards were quantified using the Accubind anti-thyroid peroxidase test system (Cat.#1125-300, Monobind Inc.). RBCs, red blood cells; L1, L2, L3; levels 1, 2, and 3 of the Liquichek Speciality Immunoassy Control, respectively

Levels of TPO-Ab in the low-, medium-, and high-quality control DBS cards were consistent with the pattern expected (see Fig. 1), indicating that measurement of TPO-Ab in DBS is feasible.

### Sensitivity and specificity of the in-house assay and determination of the DBS cut-off value for TPO-Ab positivity

Plasma samples from all 12 individuals who were positive for TPO-Ab based on the Accubind ELISA results were correctly identified as positive by the in-house assay. A plasma sample for one TPO-negative individual was incorrectly identified as positive using the in-house assay.

A cut-off value of ≥ 26 IU/mL for venous DBS (vDBS) and fingerprick DBS (fDBS) samples resulted in correct identification of all 12 individuals who were positive for TPO-Ab based on the gold standard method using vDBS samples and 11/12 positive individuals using fDBS samples. Two vDBS samples were incorrectly identified as positive for TPO-Ab whereas three of the 182 fDBS samples were incorrectly identified as positive. Sensitivity and specificity values and 95% confidence intervals are provided in Table 1.

**Table 1:** Sensitivity and specificity of the in-house TPO-Ab ELISA for 182 matched plasma, venous DBS (vDBS), and fingerprick DBS (fDBS) samples

	True positives (TP)	True negatives (TN)	False negatives (FN)	False positives (FP)	Sensitivity (%)		Specificity (%)	
					TP/(TP + FN)	95% CI	TN/(TN+ FP) (%)	95% CI
**Plasma**	12	169	0	1	100	73.5–100%	99.4	96.8–100%
**vDBS**	12	168	0	2	100	73.5–100%	98.8	95.8–99.9%
**fDBS**	11	167	1	3	91.7	61.3–99.8%	98.2	94.9–99.6%

Note that a cut-off of 26 IU/mL was used

### Characteristics of TPO-Ab-positive individuals

As specified previously, 10 of the 12 E2V2 participants positive for TPO-Ab were female, with a mean age of 33 years (range, 18–61 years). Six of these 12 individuals reported being generally ill in the 2 weeks prior to the collection date (“In the past 14 days, have you been ill (Y/N)”), and five reported difficulty with carrying out household activities. Only two of these 12 patients reported having an autoimmune disease: rheumatoid arthritis and Hashimoto’s thyroiditis, respectively.

### Limit of detection (LOD)

The LOD of the in-house assay was 1.11 IU/mL. No samples had a concentration lower than the LOD.

### Dilutional linearity

As shown in Table 2, the average percentage recovery for the 2-, 4-, and 8-fold diluted DBS samples was roughly 98%, 82%, and 85%, indicating acceptable dilutional linearity (80–120%).

**Table 2:** Dilutional linearity of the in-house TPO-Ab ELISA for dried blood spots

DBS sample		Dilution factor			
		Undiluted	2	4	8
**#1**	ng/mL	130.5	75.5	29.9	13.8
	% Expected value	**100.0**	**115.7**	**91.7**	**84.6**
**#2**	ng/mL	49.5	23.0	9.7	5.6
	% Expected value	**100.0**	**93.1**	**78.8**	**90.7**
**#3**	ng/mL	60.2	26.0	11.6	6.0
	% Expected value	**100.0**	**86.3**	**77.0**	**80.3**
**Average % recovery**			**98.4**	**82.5**	**85.2**

### Spike and recovery

The endogenous level of TPO-Ab in the unspiked pooled DBS sample was 3.01 IU/mL (negative for TPO-Ab). Spiking the sample with TPO-Ab (measured concentration in elution buffer 41.21 IU/mL, expected value 43 IU/mL) increased the concentration of the sample to 46.71, a recovery percentage of 95.7%, and this sample would now be considered positive for TPO-Ab.

### Reliability: intra- and inter-assay coefficients of variation (CV)

The average intra-assay CV for duplicate DBS punches was 5.2%. The average CV for the high DBS quality control was 11.5%, that for the medium DBS control was 14.3%, and that for the low DBS control was 24.4% (Table 3). This high CV value for the low control is not unexpected given that this measurement is sensitive to small changes in the mean when the mean value approaches zero.

**Table 3:** Calculation of inter-assay reliability of the in-house ELISA based on three DBS controls

Plate no.	High DBS control [IU/mL]	Intermediate DBS control [IU/mL]	Low DBS control [IU/mL]
1	62.9	27.7	8.8
2	62.6	24.0	13.6
3	57.1	22.1	11.2
4	53.2	21.8	9.6
5	53.0	23.0	10.1
6	56.9	19.5	6.9
7	42.9	18.3	6.7
8	53.1	18.5	8.2
**Mean**	**55.2**	**21.9**	**9.4**
**Stdev**	**6.4**	**3.1**	**2.3**
**CV**	**11.5**	**14.3**	**24.4**

### Analyte stability in DBS

**Fig. 2:**
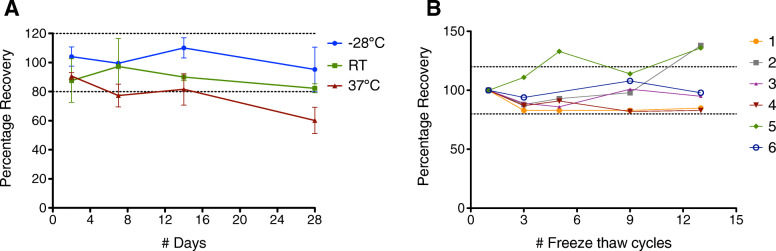
**a** Stability of thyroid peroxidase autoantibodies (TPO-Ab) concentrations in response to storage over time at different temperatures. Venous DBS (vDBS) samples from four individuals were stored at − 28 °C, at room temperature (RT, ~22 °C), and at 37 °C for 28 days. On days 2, 7, 14, and 28 of storage, samples were transferred to − 80 °C. TPO-Ab concentrations in these samples were compared to those in samples stored at − 80 °C after collection and thawed only once for this experiment. The average percentage recovery for all four samples at the various timepoints for the three different storage temperatures is shown. Error bars are standard errors of the mean. Dotted lines indicate 80 and 120% recovery. **b** Stability of TPO-Ab concentrations in response to the number of freeze-thaw cycles. vDBS samples from six individuals were stored at − 28 °C and then allowed to thaw at RT for 2 h two, four, eight, or twelve times (each on separate days) after which they were returned to the − 28 °C freezer. These samples were then thawed one final time to assay TPO-Ab concentrations, for a total number of freeze-thaw cycles of 3, 5, 8, 9, and 13, respectively. TPO-Ab concentrations in these samples were compared to those in samples stored at − 80 °C after collected and thawed only once for this experiment. Dotted lines indicate 80 and 120% recovery

A noticeable decrease in TPO-Ab levels over time was observed in samples stored at 37 °C and at room temperature over time, with the largest percentage decrease seen after storage at 37 °C for 28 days, suggesting degradation of TPO-Ab to non-immunogenic breakdown products (Fig. 2a). TPO-Ab levels of samples stored at – 28 °C were similar to those of the sample stored at – 80 °C, even after 28 days, indicating that DBS cards should be stored at – 28 °C or lower. While levels of TPO-Ab in most of the tested vDBS appeared to be stable at between 80 and 120% recovery of levels measured in the matched samples thawed only once, there was a spike in apparent TPO-Ab concentration in two samples (2 and 5) after 13 freeze-thaw cycles (Fig. 2b). It is also notable that 5/6 samples had an apparent decrease in TPO-Ab level relative to the sample thawed only once (which was set to 100% recovery).

## Discussion

We successfully developed an in-house ELISA for population-level research to assess whether an individual is positive for TPO-Ab, a biomarker of autoimmune disease, based on measurement of TPO-Ab levels in a single 3-mm DBS punch. Dilutional linearity and spike and recovery results indicated that the DBS matrix does not adversely affect the measurement of TPO-Ab levels in DBS. The intra-assay CV of 5.2% indicates acceptable intra-assay reliability. The reliability of the assay for the high and medium DBS controls was acceptable at 11.5% and 14.3%, respectively, although it should be noted that using a cut-off of 26 IU/ml, the intermediate DBS control would have misclassified as positive on plate 1, but not if it had been rerun on several plates, emphasizing the importance of rerunning samples that are near the cut-off value. Although the CV for the low control was high at 24.4%, this pattern is commonly seen in CV calculations as the mean approaches zero, and this sample would have been categorized as negative for TPO-Ab regardless of which plate it was run on. The specificity of the assay for detecting TPO-Ab in fDBS was high at 98.2% (CI 98.4–99.9%), suggesting that very few individuals that are TPO-Ab negative will be misdiagnosed as TPO-Ab positive, while the sensitivity of 91.7% (CI 61.3–99.8%) for fDBS, while high, indicates that some TPO-Ab-positive individuals may be missed. The discrepancy between the sensitivity values for the fDBS and vDBS samples may be an artifact of sample size; however, if this difference is real, it suggests some quantitative differences in the constituents of fingerprick blood and venous blood, as has been observed in previous studies [17–21].

The development of an in-house ELISA assay to assess whether an individual is TPO-positive or not using DBS samples represents a cost-effective approach that biological anthropologists can take advantage of to directly start testing the Old Friends Hypothesis in populations experiencing a more evolutionarily relevant range of parasitic infection for testing the hypothesis than those examined to date. For example, one specific hypothesis that can be tested with this DBS assay is that those individuals with a higher parasitic burden will have a lower prevalence of autoimmune antibodies [8]. In addition, this DBS assay can also be used as a screening tool for hypothyroidism in adults given that chronic autoimmune thyroiditis is the most common cause of hypothyroidism in adults in iodine-sufficient areas and that the presence of TPO-Ab usually precedes the development of thyroid dysfunction [22]. Decreased production of thyroid hormones, which are critical for normal growth and energy metabolism, adversely affects nearly all major organs, including the cardiovascular system, and if untreated can potentially be fatal [22]. In the USA, the prevalence of hypothyroidism was reported to be 4.6% based on the NHANES III study [23], but in sub-group analysis, was found to vary by the ethnic group with a higher prevalence in white and Hispanic individuals than in individuals of Afro-Caribbean descent. Higher prevalence rates than in the USA have been reported for Middle Eastern [24] and European populations [25], while lower rates have been reported in Japan [26], suggesting regional environmental differences in the development of hypothyroidism and, therefore, potentially autoimmunity. It should be noted, however, that although positivity for TPO-Ab suggests that hypothyroidism may be present, the clinical definition of hypothyroidism is based on thyroid-stimulating hormone (TSH) concentrations above the reference range and free thyroxine concentrations below the reference range; positivity for TPO-Ab together with clinical laboratory findings of elevated TSH and decrease thyroxine levels would be consistent with the diagnosis of autoimmune primary hypothyroidism [22].

A potential limitation of this DBS-based ELISA assay is that although quantitative results are obtained, only individuals with a TPO-Ab level greater than the cut-off of 26 IU/mL should be considered positive for TPO-Ab levels. This is not an issue for testing the Old Friends Hypothesis as the relevant analyses are based on the categorization of individuals as TPO-Ab positive or negative. Furthermore, for TPO-Ab positive individuals, the biological relevance of extremely high versus high or low TPO-Ab concentrations is unclear. While some authors have found no correlation between the severity of chronic autoimmune thyroiditis (Hashimoto’s thyroiditis) and anti-TPO antibody concentration [27, 28], others have found that highly elevated levels of anti-TPO antibodies are associated with a moderate increase in risk for developing hypothyroidism [29]. Further studies are needed to determine if the concentration of anti-TPO antibodies has biological relevance.

## Conclusions

In summary, we have developed a facile, non-kit-based ELISA to detect the presence of TPO-Ab in DBS. We anticipate that this will facilitate more explicit testing of the Old Friends Hypothesis across a range of populations most relevant to exploring this hypothesis. In addition, this assay could be used as an initial low-cost screening method to detect the potential presence of autoimmune thyroid disease in populations with limited access to medical facilities.

## Data Availability

Data are available upon request from the corresponding author.

## Abbreviations

AITD: Autoimmune thyroid disorder
CV: Coefficient of variation
DBS: Dried blood spot
ELISA: Enzyme-linked immunosorbent assay
fDBS: Fingerprick dried blood spot
T3: Triiodothyronine
T4: Thyroxine
TPO-Ab: Thyroid peroxidase autoantibodies
vDBS: Venous dried blood spot
